# Characteristics of Users and Nonusers of Symptom Checkers in Germany: Cross-Sectional Survey Study

**DOI:** 10.2196/46231

**Published:** 2023-06-20

**Authors:** Marvin Kopka, Lennart Scatturin, Hendrik Napierala, Daniel Fürstenau, Markus A Feufel, Felix Balzer, Malte L Schmieding

**Affiliations:** 1 Institute of Medical Informatics Charité – Universitätsmedizin Berlin, corporate member of Freie Universität Berlin and Humboldt-Universität zu Berlin Berlin Germany; 2 Division of Ergonomics Department of Psychology and Ergonomics Technische Universität Berlin Berlin Germany; 3 Institute of General Practice and Family Medicine Charité – Universitätsmedizin Berlin, corporate member of Freie Universität Berlin and Humboldt-Universität zu Berlin Berlin Germany; 4 Department of Business IT IT University of Copenhagen København Denmark

**Keywords:** symptom checker, cross-sectional study, user characteristic, digital public health, health information seeking, decision support, eHealth, mHealth, Germany, mobile health, health app, information seeking, technology use, usage, demographic, perception, awareness, adoption

## Abstract

**Background:**

Previous studies have revealed that users of symptom checkers (SCs, apps that support self-diagnosis and self-triage) are predominantly female, are younger than average, and have higher levels of formal education. Little data are available for Germany, and no study has so far compared usage patterns with people’s awareness of SCs and the perception of usefulness.

**Objective:**

We explored the sociodemographic and individual characteristics that are associated with the awareness, usage, and perceived usefulness of SCs in the German population.

**Methods:**

We conducted a cross-sectional online survey among 1084 German residents in July 2022 regarding personal characteristics and people’s awareness and usage of SCs. Using random sampling from a commercial panel, we collected participant responses stratified by gender, state of residence, income, and age to reflect the German population. We analyzed the collected data exploratively.

**Results:**

Of all respondents, 16.3% (177/1084) were aware of SCs and 6.5% (71/1084) had used them before. Those aware of SCs were younger (mean 38.8, SD 14.6 years, vs mean 48.3, SD 15.7 years), were more often female (107/177, 60.5%, vs 453/907, 49.9%), and had higher formal education levels (eg, 72/177, 40.7%, vs 238/907, 26.2%, with a university/college degree) than those unaware. The same observation applied to users compared to nonusers. It disappeared, however, when comparing users to nonusers who were aware of SCs. Among users, 40.8% (29/71) considered these tools useful. Those considering them useful reported higher self-efficacy (mean 4.21, SD 0.66, vs mean 3.63, SD 0.81, on a scale of 1-5) and a higher net household income (mean EUR 2591.63, SD EUR 1103.96 [mean US $2798.96, SD US $1192.28], vs mean EUR 1626.60, SD EUR 649.05 [mean US $1756.73, SD US $700.97]) than those who considered them not useful. More women considered SCs unhelpful (13/44, 29.5%) compared to men (4/26, 15.4%).

**Conclusions:**

Concurring with studies from other countries, our findings show associations between sociodemographic characteristics and SC usage in a German sample: users were on average younger, of higher socioeconomic status, and more commonly female compared to nonusers. However, usage cannot be explained by sociodemographic differences alone. It rather seems that sociodemographics explain who is or is not aware of the technology, but those who are aware of SCs are equally likely to use them, independently of sociodemographic differences. Although in some groups (eg, people with anxiety disorder), more participants reported to know and use SCs, they tended to perceive them as less useful. In other groups (eg, male participants), fewer respondents were aware of SCs, but those who used them perceived them to be more useful. Thus, SCs should be designed to fit specific user needs, and strategies should be developed to help reach individuals who could benefit but are not aware of SCs yet.

## Introduction

### Background

Worldwide, health experts are expecting an increasing shortage of medical personnel within the next few years [1-3]. Especially in rural areas, access to medical care is expected to decline [4]. Thus, it will become increasingly important for patients to inform themselves about their medical condition and to take the right steps based on this information. Symptom checkers (SCs) support this process of self-management [1]: these systems are defined as patient-facing decision support systems—typically using deep learning (eg, recurrent neural networks), Bayesian networks, or rule-based algorithms [5-8]—that enable laypersons to get preliminary diagnoses and recommendations for the level of care to seek based on their symptoms [8]. Like other sources of online health information [9], SCs provide health information in a convenient and scalable way.

One possible risk that emerges from the increasingly widespread usage of SCs is that they amplify existing health inequities. It is already known that racial/ethnic minorities, rural residents, and persons with a low income experience worse health care than others [10,11]. Ahmed et al [12] found that sociodemographic determinants, such as age, gender, education, and income, have an influence on the usage of electronic devices to access health information. Conversely, some authors suggest that mobile decision support systems could alleviate access problems as they provide accessible and easy-to-understand health information [13,14]. To prevent health inequities and to maximize the potential benefits of SCs, it is crucial to understand the factors that contribute to people using and not using them.

SCs commonly offer 2 features to their users: (1) Users can improve their self-diagnosis by obtaining a rank-ordered list of the most likely diagnoses, and (2) SCs can be used to assist with triage decisions. This means that they advise patients on whether it is necessary to seek care at all and, if so, how urgently (eg, instantly or within some days) they should visit which health care facility (eg, emergency department or general practitioner) [15]. Especially, the accuracy and safety of this triage function is an ongoing topic of concern for both patients and health care professionals. Their performance seems to be mediocre on average, with high variability between them [16,17].

### Related Work

Prior related studies have mainly focused on the effects of sociodemographic factors on the intention to use, trust in, or adherence to decision support systems in general. Age, gender, the level of education, and several individual factors have been already found to influence the interaction with and usage of SCs specifically. These findings will be briefly summarized next.

Users of SCs tend to be younger (with a mean age of about 40 years), and the willingness to use SCs seems to decline with increasing *age* [18-22]. However, younger users seem to find SCs more useful, but older users have been found to be more likely to recommend them [19,23].

Users also seem more commonly to be female (estimates range from 62% to 85%), although gender has also been reported to not impact the willingness to use such tools [19,20,22].

Lastly, users of SCs tend to have higher levels of formal education, which is associated with an increased likelihood of searching for diagnoses online [8,20,24-27].

In addition to sociodemographic factors, previous studies have found that people with higher *eHealth literacy* are more inclined to use mobile health apps in general and that a lack of computer literacy seems to be one of the greatest barriers to using SCs [22,28,29].

Another relevant interindividual trait is trust, as incorrect diagnoses and increased anxiety are the main concerns when using SCs [22,30]. Thus, (the propensity to) *trust* is found to impact the interaction with SCs as well [31].

Most quantitative findings on SC users stem to date from studies investigating samples using a single SC only. Although users generally perceive SCs as useful [32,33], a lack of awareness of these tools has been discussed as a potential barrier to broader adoption [34].

### Objective

The aim of this work is to refine our understanding of how sociodemographic and interindividual characteristics influence the awareness, usage, and perceived usefulness of SC apps. In contrast to most of the previous research that is based on UK, US, or Canadian users of specific SCs (which might not be representative for SC users in general), we investigated a representative sample of German-speaking internet users. Building upon the existing literature, our paper focuses on factors previously shown to be relevant in other countries. Unlike most previous studies on SC users, we sampled not only users but also nonusers of SCs. This broader sampling approach allowed us to address more questions, for example, investigating the potential reasons for unequal usage of SCs across sociodemographic factors. Not being limited to the user group of a specific SC, our approach also yielded more generalizable findings concerning factors influencing usage in the population.

## Methods

### Study Design, Participants, and Sampling

We conducted a cross-sectional online survey among German residents between July 15 and 26, 2022. Our aim was to sample 1000 participants. No prior sample size calculation was conducted, as the sample size was ultimately determined by the available budget. Considering that some participants were expected to respond incorrectly to control questions, we planned to oversample by 10% (resulting in about 1100 participants). Stratified random sampling was used to sample participants using the ISO 26362–certified sampling provider Bilendi/respondi [35]. We stratified the sample by gender, federal state, income, and age to reflect the German population [36]. Bilendi/respondi was selected because it is a commercial provider that is certified, offers panel surveys with stratified random samples, and has been used by other authors for surveying nationally representative samples in biomedical research [37-39].

The study included participants who were at least 18 years old, and excluded underage participants and those who refused to consent. Moreover, we excluded data from analysis if a participant answered one of the embedded control questions incorrectly. Upon completion, participants received a payment of EUR 1.00 (US $1.08) for their participation.

### Ethical Considerations

This study was approved by the Ethics Committee of the Charité – Universitätsmedizin Berlin (EA4/018/22). Prior to enrollment, participants provided informed consent and volunteered to take part in the survey. The study was conducted and reported according to the Checklist for Reporting Results of Internet E-Surveys (CHERRIES) guideline [40].

### Survey and Instruments

We developed a survey in German and administered it as an online questionnaire using the Unipark EFS Survey [41]. The authors and a sufficiently large convenience sample (N=9) [42] from the authors’ personal and professional network conducted pretests of the survey to ensure comprehensibility of the questions and usability of the online survey and identify any technical issues. We rearranged the survey sections and simplified the language of the questions following the pretest. All collected data were stored in EFS Survey accessible only to the authors. Participants filled out the survey remotely upon an invitation from the sampling provider, and they were prevented from participating more than once by assessing their pseudonymized ID assigned by Bilendi/respondi. The assigned ID was not shared with the authors.

Overall, the survey had 4 sections: (1) sociodemographic and interindividual characteristics, (2) questions about previously received diagnoses and medical care, (3) the usage of technology and health apps in general, and (4) the usage of SC apps in particular.

The questions about demographics and characteristics included age, gender, the level of formal education, the federal state (Bundesland) participants reside in, the municipality size, the disposable income (assessed using the Organisation for Economic Co-operation and Development [OECD]–modified scale [43]), their migration background, and their self-efficacy (measured using the Allgemeine Selbstwirksamkeit Kurzskala, ASKU, [44]).

In the second section (diagnoses and medical care), we asked participants to fill out the Minimum European Health Module (MEHM) [45] to rate their self-perceived health, including activity limitations and chronic morbidity. We also presented them with a selection of different diseases (for which officially approved health apps are available in Germany), and they could choose all diagnoses that applied to them, including depression, panic or anxiety disorder, and chronic pain. Health care usage was assessed by asking for the insurance type (statutory, private, other, or none), whether they have a permanent general practitioner (yes/no), how often they visited a general practitioner within the last 12 months (open numerical text field), whether they are undergoing psychotherapy, and whether they have been hospitalized as an inpatient in the past 12 months (yes/no). We included psychotherapy in our definition of health care usage as the German statutory health insurances cover mental health services and many digital health apps, including SCs, are sought for psychiatric or psychosomatic issues.

In the third section, we asked participants how often they use the internet (several times a day, once a day, several times a week, several times a month, or less than once a month) and we assessed their affinity for technology interaction (using the Affinity for Technology Interaction [ATI] scale [46]). We also assessed their health app usage by asking whether they generally use health apps (yes/no).

In the last section, we gave participants a description of SCs and asked them about SCs using 3 steps: First, we asked whether they know about SCs (yes/no). If they affirmed, they were asked whether they had used them before (yes/no). If they did, we asked them to rate their usefulness on a 5-point Likert scale with the levels 1=“not useful at all,” 2=“rather not useful,” 3=“sometimes useful, sometimes not,” 4=“rather useful,” and 5=“very useful.”

We embedded 2 control questions in the questionnaire asking participants to select a particular answer option to a mock question (eg, “Please select ‘does not apply’”).

### Data Analysis

The data were analyzed exploratively—all values (including *P* values along with other measures of statistical inference) should therefore be interpreted in a hypothesis-generating manner. We included robustness checks (see Multimedia Appendix 1) adjusting *P* values for multiple testing using the Benjamini-Hochberg procedure to verify that the results remained valid after correction. Our significance level was set to .05. For income, we controlled for unreasonable data by excluding outliers (defined as the top 2.5% and the bottom 2.5% income).

First, we compared those aware of SCs and those unaware of them. Second, we compared SC users with nonusers, (1) in all participants and (2) in a subset of those being aware of SCs, to assess factors that may contribute to the willingness to use. Lastly, to assess factors influencing the perceived usefulness of SCs, we included only data from participants who had used SCs before. We divided usefulness into “not useful” (indicated by selecting “not useful at all” or “rather not useful”), a middle category (“sometimes useful, sometimes not”), and “useful” (indicated by selecting “rather useful” or “very useful”).

We conducted comparative analyses of these subsets by comparing all characteristics using summary statistics (mean and SD for metric variables, absolute numbers, percentages, and 95% CIs for binary, multinomial, and ordinal variables). For inferential analyses, we used Welch *t* tests (for metric variables with groups of different sample sizes); chi-square tests (for binary and multinomial variables), or Fisher exact tests when any cell contained less than 5 observations; and Mann-Whitney U tests (for ordinal variables). To quantify effect sizes, we used Cohen d for *t* tests, the phi coefficient (φ) for 2×2 chi-square tests/Fisher exact tests, Cramer V for more than 2×2 chi-square tests/Fisher exact tests, and the Glass rank biserial correlation coefficient rg for Mann-Whitney U tests. Further, we visualized selected characteristics in raincloud plots [47].

In Multimedia Appendix 1, we provide the following additional analysis: To explore perceived usefulness in more detail, we correlated usefulness with other binary (point-biserial correlation) and continuous variables (Pearson correlation) and visualized it in a heatmap using 1 column of a correlation matrix.

We used R version 4.1.2 [48] and the *tidyverse* packages [49] to manipulate and analyze the collected data. We also used the packages *rstatix* [50] to compute summary statistics and correlation matrices; *DAAG* [51] to assess the variance inflation factors; *ggdist* [52] and *gghalves* [53], in addition to *ggplot2* [54], for data visualization; and *DescTools* [55] to compute CIs. For effect size computation, we used *rstatix* (Cohen d), *rcompanion* (Glass rank biserial correlation coefficient rg) [56], *DescTools* (Cramer V), and the *psych* package (φ) [57].

To make the Results section more concise, mostly statistically significant results are reported in tables summarizing group comparisons of participant characteristics. Detailed tables outlining all findings and inferential statistics are provided in Multimedia Appendix 1.

## Results

### Participants

A total of 1555 people accessed the survey, of which 400 (25.7%) did not complete it. Moreover, 4 (0.3%) participants were screened out: 2 (50.0%) for indicating to be younger than 18 years and 2 (50.0%) for not providing informed consent. We excluded the data of 67 (4.3%) participants due to incorrect answers to at least 1 of 2 control questions. As a result, we included the data of 1084 (69.7%) participants in our study. About 1 in 6 participants (177/1084, 16.3%) indicated having previously heard about SCs. Of these, 40.1% (71/177)—equating to 6.5% (71/1084) of the total sample—reported having used an SC at least once before. Participants’ characteristics (with all collected variables) are shown in Table 1.

**Table 1:** Characteristics of respondents (N=1084).

Characteristics		Respondents
Age (years), mean (SD)		46.7 (15.9)
**Gender, n (%)**		
	Male	521 (48.0)
	Female	560 (51.7)
	Diverse	3 (0.3)
**Education, n (%)**		
	No school diploma	5 (0.5)
	Primary school/lower secondary school	61 (5.6)
	Secondary school leaving certificate	225 (20.8)
	A level/high school diploma	166 (15.3)
	Completed vocational training	317 (29.2)
	University or college degree	310 (28.6)
Monthly net household income (EUR/US $^a^), mean (SD)		1868.82 (894.45)/2018.33 (966.01)
**Municipality size, n (%)**		
	<5000	178 (16.4)
	5000-10,000	129 (11.9)
	10,000-20,000	146 (13.5)
	20,000-50,000	168 (15.5)
	50,000-100,000	101 (9.3)
	100,000-500,000	196 (18.1)
	>500,000	166 (15.3)
Migration background, n (%)		123 (11.3)
Native German speaker, n (%)		1044 (96.3)
Self-efficacy, mean (SD)^b^		3.96 (0.72)
**General health, n (%)**		
	Very bad	15 (1.4)
	Bad	97 (8.9)
	Fair	301 (27.8)
	Good	540 (49.8)
	Very good	131 (12.1)
**Restrictions for health reasons, n (%)**		
	Not limited at all	459 (42.3)
	Limited but not severely	475 (43.8)
	Severely limited	150 (13.8)
**Diagnosis, n (%)**		
	Chronic disease	544 (50.2)
	Depression	166 (15.3)
	Panic or anxiety disorder	110 (10.1)
	Chronic pain	141 (13.0
**Type of health insurance, n (%)**		
	Without health insurance	4 (0.4)
	Statutory health insurance	958 (88.4)
	Private health insurance	114 (10.5)
	Other	7 (0.6)
Permanent general practitioner, n (%)		984 (90.8)
Number of physician visits in the past year, mean (SD)		3.87 (6.15)
In psychotherapy, n (%)		93 (8.7)
At least 1 inpatient hospital stay in the past year, n (%)		171 (15.8)
**Frequency of internet use, n (%)**		
	Multiple times a day	996 (91.9)
	Once a day	65 (6.0)
	Multiple times a week	19 (1.8)
	Multiple times a month	2 (0.2)
	Less than once a month	2 (0.2)
Affinity for technology, mean (SD)^c^		3.74 (1.0)
General health app usage, n (%)		517 (48.5)

^a^EUR 1.00=US $1.08.

^b^On a scale of 1-5.

^c^On a scale of 1-6.

### Comparison Between Participants Aware and Unaware of SC Apps

Participants aware of SCs were commonly younger (mean 38.8, SD 14.6 years, vs mean 48.3, SD 15.7 years; *P*<.001), were more commonly female (107/177, 60.5%, vs 453/907, 49.9%; *P*=.015), had higher formal education levels (eg, 72/177, 40.7%, vs 238/907, 26.2%, with a university r college degree; *P*<.001), and on average reported a higher net household income (mean EUR 2173.96, EUR SD 992.83 [mean US $2347.88, SD US $1072.26], vs mean EUR 1813.79, SD EUR 865.37 [mean US $1958.89, SD US $934.60]; *P*<.001) than the remaining study participants. About three-quarters of the participants being aware of SCs (128/177, 74.0%) reported prior experience of using health apps in general in contrast to the remaining participants, of which less than half reported this (389/907, 43.5%; *P*<.001). They also showed higher scores on the ATI scale (mean 4.17, SD 0.93, vs mean 3.66, SD 0.99; *P*<.001). We found only little differences between these groups regarding self-reported general health and migration background. A summary of all characteristics and interindividual differences between those who knew SCs and those who did not is provided in Table 2 and Table S1 in Multimedia Appendix 1.

**Table 2:** Characteristics of and interindividual differences between respondents aware and not aware of SCs^a^.

Characteristics		Aware of SCs	Not aware of SCs
Participants, n (%); 95% CI		177 (16.3); 14.2%-18.7%	907 (83.7); 81.3%-85.8%
Age (years), mean (SD)		38.8 (14.6)	48.3 (15.7)
**Gender, n (%); 95% CI**			
	Male	69 (39.0); 32.2%-46.8%	452 (49.8); 46.5%-53.3%
	Female	107 (60.5); 53.7%-68.3%	453 (49.9); 46.6%-53.4%
	Diverse	1 (0.6); 0.0%-8.4%	2 (0.2); 0.0%-3.7%
**Education, n (%); 95% CI**			
	No school diploma	0 (0.0); 0.0%-7.8%	5 (0.6); 0.0%-4.0%
	Primary school/lower secondary school	7 (4.0); 0.0%-11.8%	54 (6.0); 2.5%-9.4%
	Secondary school leaving certificate	27 (15.3); 7.9%-23.1%	198 (21.8); 18.4%-25.3%
	A level/high school diploma	35 (19.8); 12.4%-27.6%	131 (14.4); 11.0%-17.9%
	Completed vocational training	36 (20.3); 13.0%-28.2%	281 (31.0); 27.6%-34.4%
	University or college degree	72 (40.7); 33.3%-48.5%	238 (26.2); 22.8%-29.7%
Monthly net household income (EUR/US $^b^), mean (SD)		2173.96 (992.83)/2347.88 (1072.26)	1813.79 (865.37)/1958.89 (934.60)
**General health,** **n (%)** **; 95% CI**			
	Very bad	2 (1.1); 0.0%-8.9%	13 (1.4); 0.0%-4.9%
	Bad	10 (5.6); 0.0%-13.4%	87 (9.6); 6.3%-13.1%
	Fair	43 (24.3); 16.9%-32.1%	258 (28.4); 25.1%-31.9%
	Good	89 (50.3); 42.9%-58.1%	451 (49.7); 46.4%-53.2%
	Very good	33 (18.6); 11.3%-26.4%	98 (10.8); 7.5%-14.3%
Panic or anxiety disorder, n (%); 95% CI		27 (15.3); 10.7%-21.3%	83 (9.2); 7.4%-11.2%
**Type of health insurance, n (%); 95% CI**			
	Without health insurance	0 (0); 0.0%-5.4%	4 (0.4); 0.0%-2.4%
	Statutory health insurance	147 (83.1); 78.0%-88.5%	811 (89.5); 87.5%-91.4%
	Private health insurance	29 (16.4); 11.3%-21.8%	85 (9.4); 7.5%-11.3%
	Other	1 (0.5); 0.0%-6.0%	6 (0.7); 0.0%-2.6%
Permanent general practitioner, n (%); 95% CI		153 (86.4); 80.6%-90.7%	831 (91.6); 89.6%-93.3%
In psychotherapy, n (%); 95% CI		25 (14.2); 9.8%-20.1%	68 (7.5); 6.0%-9.5%
Affinity for technology, mean (SD)^c^		4.17 (0.93)	3.66 (0.99)
General health app usage, n (%); 95% CI		128 (74.0); 67.0%-80.0%	389 (43.5); 40.3%-46.8%

^a^SC: symptom checker.

^b^EUR 1.00=US $1.08.

^c^On a scale of 1-6.

### Comparison Between Participants Using and Not Using SC Apps

First, we present results comparing users and all other participants, allowing us to compare the characteristics of users and the general public. To assess the inclination to use SCs, we contrasted the characteristics of users and nonusers in the subset of participants who were aware of these tools.

#### Comparison Between SC Users and All Remaining Participants

**Figure 1:**
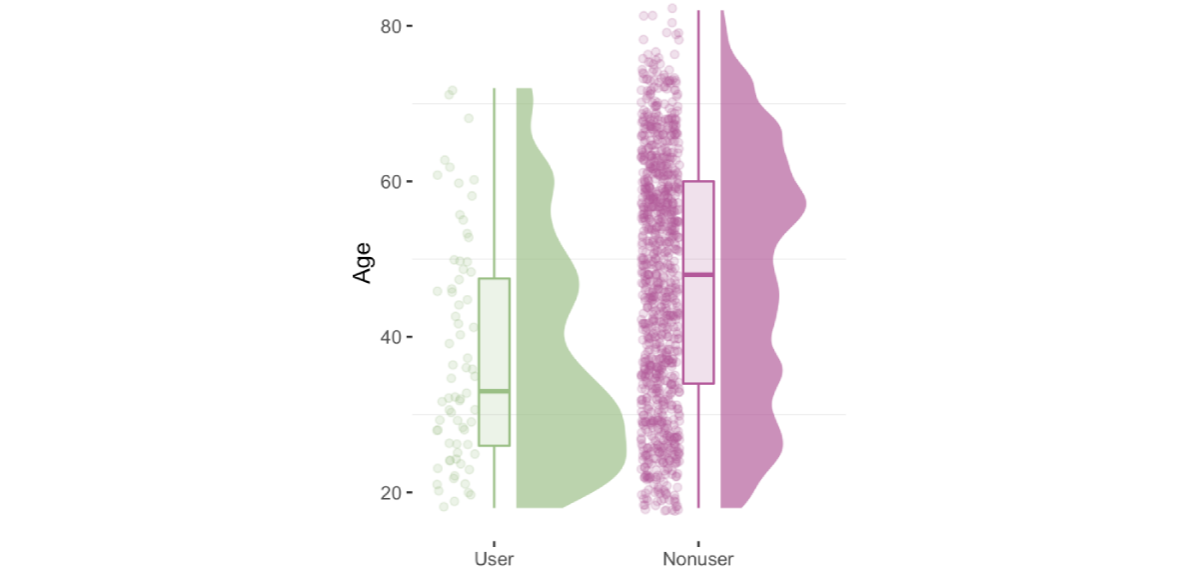
Age distribution of SC users and nonusers. SC: symptom checker.

Compared to all nonusers (mean 47.3, SD 15.8), SC users were younger (mean 37.6, SD 14.3 years; *P*<.001; see Figure 1 and Table 3) and more likely to be female (44/71, 62.0%, vs 516/1013, 50.9%; *P*=.030). SC users also had a higher level of formal education: 84.5% (60/71) had a high school diploma, had completed vocational training, or had a university degree compared to 72.4% (733/1013) of nonusers (*P*=.035). SC users also reported a higher net household income on average (mean EUR 2248.18, SD EUR 1052.60 [mean US $2428.03 SD US $1136.81], vs mean EUR 1841.16, SD EUR 876.04 [mean US $1988.45, SD US $946.12]; *P*=.002). They more commonly indicated restrictions due to health reasons (54/71, 76.1%, vs 571/1013, 56.3%; *P*=.002) and to suffer from a mental illness (29/71, 40.8%, vs 197/1013, 19.4%; *P*<.001; see Table 3). SC users had a higher affinity for technology (mean 4.13, SD 0.95) compared to nonusers (mean 3.72, SD 1.00; *P*<.001) and more commonly used other health apps (61/71, 87.1%, vs 456/1013, 45.7%; *P*<.001). Table 3 and Table S2 in Multimedia Appendix 1 provide additional characteristics and interindividual differences.

**Table 3:** Characteristics of and interindividual differences between SC^a^ users and nonusers.

Characteristics		Users	Nonusers
Participants, n (%); 95% CI		71 (6.5); 5.2%-8.2%	1013 (93.5); 91.8%-94.9%
Age (years), mean (SD)		37.6 (14.3)	47.3 (15.8)
**Gender, n (%); 95% CI**			
	Male	26 (36.6); 26.8%-49.0%	495 (48.9); 45.7%-52.1%
	Female	44 (62.0); 52.1%-74.4%	516 (50.9); 47.8%-54.2%
	Diverse	1 (1.4); 0.0%-13.8%	2 (0.2); 0.0%-3.5%
**Education, n (%); 95% CI**			
	No school diploma	0 (0); 0.0%-12.2%	5 (0.5); 0.0%-3.8%
	Primary school/lower secondary school	4 (5.6); 0.0%-17.8%	57 (5.6); 2.5%-9.0%
	Secondary school leaving certificate	7 (9.9); 0.0%-22.0%	218 (21.5); 18.4%-24.9%
	A level/high school diploma	14 (19.7); 8.5%-31.9%	152 (15.0); 11.8%-18.3%
	Completed vocational training	16 (22.5); 11.3%-34.7%	301 (29.7); 26.6%-33.1%
	University or college degree	30 (42.3); 31.0%-54.4%	280 (27.6); 24.5%-31.0%
Monthly net household income (EUR/US $^b^), mean (SD)		2248.18 (1052.60)/2428.03 (1136.81)	1841.16 (876.04)/1988.45 (946.12)
**Restrictions for health reasons, n (%); 95% CI**			
	Not limited at all	17 (23.9); 12.7%-35.7%	442 (43.6); 40.4%-47.0%
	Limited but not severely	40 (56.3); 45.1%-68.1%	435 (42.9); 39.7%-46.3%
	Severely limited	14 (19.7); 8.5%-31.5 %	136 (13.4); 10.2%-16.8%
**Diagnosis, n (%); 95% CI**			
	Depression	22 (31.0); 21.4%-42.5%	144 (14.2); 12.2%-16.5%
	Panic or anxiety disorder	16 (22.5); 14.4%-33.5%	94 (9.3); 7.6%-11.2%
Currently undergoing psychotherapy, n (%); 95% CI		18 (25.4); 16.7%-36.6%	75 (7.5); 5.9%-9.2%
At least 1 inpatient hospital stay in the past year, n (%); 95% CI		21 (29.6); 20.2%-41.0%	150 (14.8); 12.8%-17.1%
Affinity for technology, mean (SD)^c^		4.13 (0.95)	3.72 (1.00)
General health app usage, n (%); 95% CI		61 (85.9); 76.0%-92.2%	456 (45.7); 42.0%-48.1%

^a^SC: symptom checker.

^b^EUR 1.00=US $1.08.

^c^On a scale of 1-6.

#### Comparison Between SC Users and Nonusers Aware of SCs

When comparing SC users (n=71, 6.5%) with the remaining participants who were aware of SCs but without prior experience using them (n=106, 9.8%), some differences remained, while others disappeared: age (mean 37.6, SD 14.3 years, vs mean 39.6, SD 14.8 years; *P*=.380), gender (*P*=.464), and net household income distribution were similar between these groups (mean EUR 2248.18, SD EUR 1052.60 [mean US $2428.03, SD US $1136.81], vs mean EUR 2122.00, SD EUR 951.70 [mean US $2291.76, SD US $1027.84]; *P*=.425). Affinity for technology was about equal (mean 4.13, SD 0.95, vs mean 4.19, SD 0.91; *P*=.627), too, and similar to the comparison of users and nonusers, self-efficacy was not associated with the awareness or usage of SC apps.

New differences between these groups appeared regarding health-related factors: Users reported worse general health. Of the 71 users, 6 (8.4%) reported very bad or bad health compared to 6/106 (5.6%) nonusers, 7 (9.9%) reported very good health compared to 26/106 (24.5%) nonusers (*P*=.009), and 54 (76.1%) reported more health-related restrictions compared to 53/106 (51.9%) nonusers (*P*=.001); in addition, users reported more frequent physician visits (mean 4.51, SD 3.69) compared to nonusers (mean 3.08, SD 3.81; *P*=.014). We found the rate of SC usage to be higher among those self-reporting depression (22/71, 31.0%, vs 9/106, 8.5%; *P*<.001), self-reporting panic or anxiety disorder (16/71, 22.5%, vs 11/106, 10.4%; *P*=.046), and undergoing psychotherapy (18/71, 25.4%, vs 7/106, 6.6%; *P*=.001). Tabular and graphical summaries of sociodemographic characteristics and interindividual differences between users and nonusers aware of SCs are provided in Table 4, Table S3 in Multimedia Appendix 1, and Figure S2 in Multimedia Appendix 1.

**Table 4:** Characteristics of and interindividual differences between SC^a^ users and nonusers among respondents aware of SCs.

Characteristics		Using SCs	Aware of but not using SCs
Participants, n (%); 95% CI		71 (40.1); 32.8%-47.7%	106 (59.9); 52.3%-67.2%
**General health, n (%); 95% CI**			
	Very bad	1 (1.4); 0.0%-13.5%	1 (0.9); 0.0%-11.0%
	Bad	5 (7.0); 0.0%-19.2%	5 (4.7); 0.0%-14.7%
	Fair	22 (31.0); 19.7%-43.1%	21 (19.8); 10.4%-29.8%
	Good	36 (50.7); 39.4%-62.8%	53 (50.0); 40.6%-60.0%
	Very good	7 (9.9); 0.0%-22.0%	26 (24.5); 15.1%-34.5%
**Restrictions for health reasons, n (%); 95% CI**			
	Not limited at all	17 (23.9); 12.7 –35.7%	53 (50.0); 40.6%-60.2%
	Limited but not severely	40 (56.3); 45.1%-68.1%	40 (39.6); 28.3%-47.9%
	Severely limited	14 (19.7); 8.5%-31.5 %	13 (12.3); 2.8%-22.5%
**Diagnosis, n (%); 95% CI**			
	Chronic disease	41 (57.7); 46.2%-68.5%	42 (39.6); 30.8%-49.1%
	Depression	22 (31.0); 21.4%-42.5%	9 (8.5); 4.5%-15.4%
	Panic or anxiety disorder	16 (22.5); 14.4%-33.5%	11 (10.4); 5.9%-17.6%
Permanent general practitioner, n (%); 95% CI		67 (94.4); 86.4%-97.8%	86 (81.1); 72.6%-87.4%
Number of physician visits in the past year, mean (SD)		4.51 (3.69)	3.08 (3.81)
Currently undergoing psychotherapy, n (%); 95% CI		18 (25.4); 16.7%-36.6%	7 (6.6); 3.2%-13.0%
At least 1 inpatient hospital stay in the past year, n (%); 95% CI		21 (29.6); 20.2%-41.0%	15 (14.2); 8.8%-22.0%
General health app usage, n (%); 95% CI		61 (85.9); 76.0%-92.2%	67 (63.2); 53.7%-71.8%

^a^SC: symptom checker.

### Usefulness of SCs

Of the 71 users, 29 (40.8%) considered SCs (rather) useful, while 18 (25.4%) found them (rather) not useful. The remaining one-third of participants (24/71, 33.8%) reported that SCs were sometimes useful and sometimes not useful to them.

Between users considering SCs useful and those who did not (disregarding those who found them sometimes useful and sometimes not), all sociodemographic variables except for age revealed differences; see Table 5. In our sample, males were 4 times (16:4) more likely than females (13:13; odds ratio [OR] 4.0) to rate their experience with SCs as useful. The difference in general usefulness was statistically significant (*P*=.002). A higher net household income was also strongly associated with usefulness (mean EUR 2591.63, SD EUR 1103.96 [mean US $2798.96, SD US $1192.28], among those considering SCs useful vs mean EUR 1626.60, SD EUR 649.05 [mean US $1756.73, SD US $700.97], among those who did not; *P*<.001). Additionally, a higher level of formal education was found among participants rating the usefulness of SCs favorably (55.2% vs 22.2% with a university or college degree; *P*=.016).

**Figure 2:**
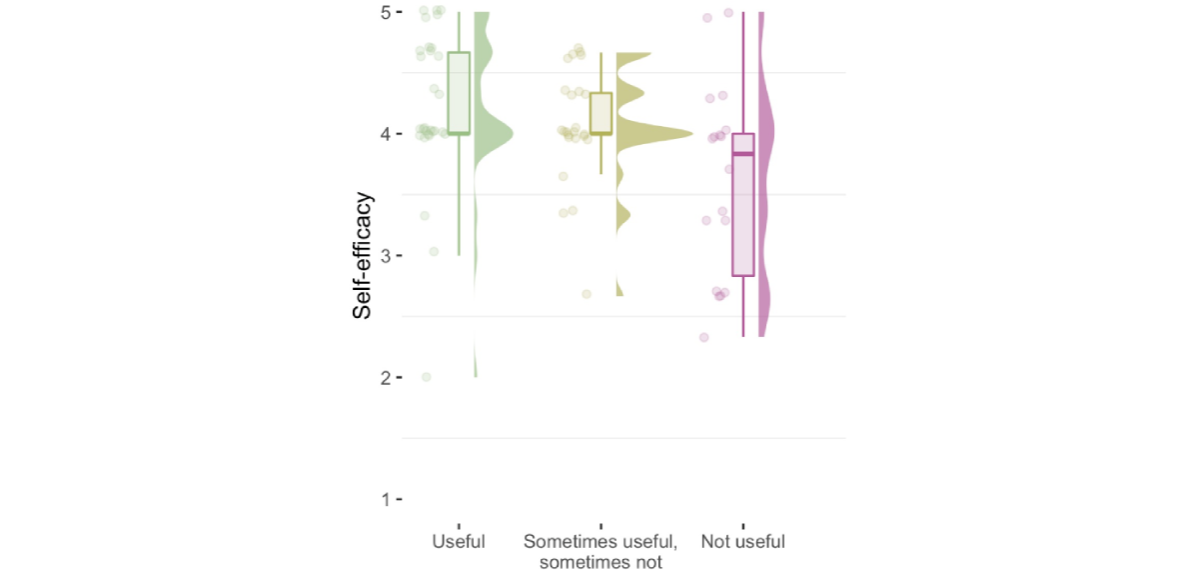
Self-efficacy by usefulness rating. Above a certain threshold of self-efficacy, users are about equally likely to rate the app as useful or not useful, but below that threshold, they commonly find it unhelpful.

Higher self-efficacy scores were also associated with rating SC usage as useful: Visual analysis of the association between self-efficacy and usefulness hinted at a threshold effect; see Figure 2. Although users with a self-efficacy score above 3.5 commonly found SCs useful (25:10), users below this threshold did not (3:8; OR 6.6). Similarly, most participants (9:1) scoring very high (>5/6) on the ATI scale considered SCs useful, while the majority (3:4) of users with a low score (<3/6) did not. Mean and median scores for affinity for technology, however, were similar across these 2 groups (see Figure S1 in Multimedia Appendix 1). Although in lesser magnitude, these observations held when including participants rating their previous experience as “sometimes helpful, sometimes unhelpful.”

Although users rating their experience as useful self-reported higher general health and lower restrictions for health reasons than users with unhelpful experiences with SCs, these findings were not statistically significant and had a small effect size. Rates of all 3 indicators of health care usage (inpatient hospital stay within the past year, number of physician visits within the past year, currently undergoing psychotherapy) were higher among participants considering SCs useful. In contrast, users currently suffering from panic or anxiety disorder more commonly considered SCs not useful than useful (see Table S4 in Multimedia Appendix 1).

Although the usage of other health apps in general was strongly associated with the awareness and usage of SCs, it was not associated with considering SCs useful.

Table 6 summarizes the gender distribution of participants who were aware of SCs, used them, and considered them useful: women seemed to know and use SCs more frequently. However, the proportion of users among those who knew about SCs was similar for men and women, but men found SCs more commonly useful.

**Table 5:** Characteristics of and interindividual differences between users considering SCs^a^ useful and not useful.

Characteristics		Considered SCs useful	Considered SCs sometimes useful, sometimes not	Did not consider SCs useful
Participants, n (%); 95% CI		29 (40.8); 29.3%-53.2%	24 (33.8); 23.0%-46.0%	18 (25.4); 15.8%-37.1%
**Gender, n (%); 95% CI**				
	Male	16 (55.2); 41.4%-75.8%	6 (25.0); 12.5%-43.9%	4 (22.2); 5.6%-42.4%
	Female	13 (44.8); 31.0%-65.5%	18 (75.0); 62.5%-93.9%	13 (72.2); 55.6%-92.4%
	Diverse	0 (0.0); 0.0%-20.6%	0 (0.0); 0.0%-18.9%	1 (5.6); 0.0%-25.7%
**Education, n (%); 95% CI**				
	No school diploma	0 (0.0); 0.0%-19.7%	0 (0.0); 0.0%-22.3%	0 (0.0); 0.0%-27.7%
	Primary school/lower secondary school	2 (6.9); 0.0%-26.6%	1 (4.2); 0.0%-26.45%	1 (5.6); 0.0%-33.2%
	Secondary school leaving certificate	4 (13.8); 0.0%-33.5%	3 (12.5); 0.0%-34.8%	0 (0.0); 0.0%-27.7%
	A level/high school diploma	3 (10.3); 0.0%-30.1%	5 (20.8); 4.2%-43.1%	6 (33.3); 16.7%-61.0%
	Completed vocational training	4 (13.8); 0.0%-33.5%	5 (20.8); 4.2%-43.1%	7 (38.9); 22.2%-66.5%
	University or college degree	16 (55.2); 41.4%-74.9%	10 (41.7); 25.0%-64.0%	4 (22.2); 5.6%-49.9%
Monthly net household income (EUR/US $^b^), mean (SD)		2591.63 (1103.96)/2798.96 (1192.28)	2273.47 (1054.62)/2455.35 (1138.99)	1626.60 (649.05)/1756.73 (700.97)
Self-efficacy, mean (SD)^c^		4.21 (0.66)	4.07 (0.48)	3.63 (0.81)

^a^SC: symptom checker.

^b^EUR 1.00=US $1.08.

^c^On a scale of 1-5.

**Table 6:** Gender^a^ distribution of those knowing about SCs^b^, using them, the proportion of users among those knowing about them, and their usefulness rating.

Subgroup	Male	Female
Know about SCs, n/N (%)	69/521 (13.2)	107/560 (19.1)
Use SCs, n/N (%)	26/521 (5.1)	44/560 (7.7)
Proportion of users among people knowing about SCs, n_User_/n_Knowing_ (%)	26/69 (37.7)	44/107 (41.1)
Proportion of users finding SCs either “rather useful” or “very useful,” n_useful_/n_users_ (%)	16/26 (61.5)	13/44 (29.6)

^a^Due to the low sample size (n=3, 0.3%), participants of a diverse gender are not reported here.

^b^SC: symptom checker.

## Discussion

### Principal Findings

#### Prevalence of SC Usage

Our cross-sectional survey found that only a minority of 16.3% of German people with internet access are aware of SC apps, and among them, only a minority of 40.1% report having used SCs at least once before. Thus, we find the proportion of SC users among the German online population to be lower (6.5%) than the figure of diagnostic app users (13.0%) previously reported [58].

The low prevalence of SC usage suggests that the current users are “innovators” and “early adopters,” as defined by diffusion of innovation theory [59].

#### Misalignments in Subgroups

Comparing subgroups of participants, we identified sociodemographic and other interindividual characteristics associated with knowing about and using SCs and considering them useful. Taking these findings together, our study hints at some misalignments between factors associated with using and benefiting from SCs, that is, there are some groups that might potentially benefit from these tools but are less inclined to use them and others that are inclined to use them but often do not benefit from them (eg, those with panic or anxiety disorder).

##### Gender

A prime example of this misalignment in our data is gender: our study concurs with previous research that women more commonly use SCs than men [19,20]. At the same time, men who are aware of SCs are about equally likely to use SCs as women, which concurs with the Healthwatch Enfield study [22]. Taken together, this suggests that this gender gap is not due to dissimilar conversion rates.

Similarly, gender-specific differences in the perception of the usefulness of SCs do not seem a plausible driver of unequal gender usage either, as among users, men more commonly reported considering SCs useful than their female counterparts. Thus, the disparity in the awareness of SCs between men and women might be the primary cause behind the gender gap in usage. A multitude of reasons might explain that effect: Women are more often responsible for care work [60] and, therefore, potentially more likely to search for online health information on someone else’s behalf. Women may also seek health information more often (and find SCs) because of higher health anxiety [61]. Third, advertisements from SC developers might be directed more toward women than men.

Due to the small sample size of SC users, we can only speculate as to why men more often considered their usage to have been helpful: As previously published studies suggest men being less risk-averse than SCs (and women) regarding triage decisions [17,62], a differing second opinion might be considered more useful than a confirmative one. Additionally, as women are more inclined to inform themselves about symptoms, health topics, and the health care system [63-66], the additional benefit from SCs might be less pronounced.

##### Age

Regarding age, we found a similar pattern as for gender. Although SC users were younger than nonusers (in line with previous research [18-20,23]), we found no association between age and the inclination to use SCs in the group that was aware of SCs, in contrast to the Healthwatch Enfield study [22]. Furthermore, age was not associated with perceived usefulness, as reported elsewhere [19]. Thus, older patients may also benefit from SCs when informed about these tools. As the amount of care required increases with age [67], the potential of SCs for older patients and how they can become aware of them should be a priority for further investigation.

##### Education

Like other studies [20,34], we found SC users to have a high level of formal education. Formal education followed a comparable pattern as age and gender: Participants with higher levels of formal education were more commonly aware of SCs, but once participants were aware, the level of education did not influence the inclination to use or self-reporting of benefits from SCs.

##### Income

Factors showing relevant but distinct patterns were income, health-related variables, affinity for technology, and self-efficacy. A higher income was associated with both a higher awareness of SCs and greater perceived usefulness. Although the higher awareness might again be a result of the marketing strategy of SC developers, the limited number of SC users in our sample allows no conclusive argumentation as to why higher-income users reported to benefit more from SCs. As income is a function of a combination of different socioeconomic factors and interindividual traits, higher-income users might approach SCs for different reasons and with different expectations than lower-income users.

##### Health Status

Concerning health-related variables, prior usage of health apps correlates highly with the awareness of SCs and the inclination to use them but is not a predictor of finding the usage beneficial. Thus, akin to age, gender, and formal education, people previously unaware of health apps might benefit from them once they are aware of them. Because SC users commonly tend to be younger, have a high income, and are well-educated individuals, one may conclude that SCs cater to a healthier subgroup of the population. However, our findings show no such health gap: users and nonusers appraised their self-reported general health level equally. The reported restrictions in daily life due to health-related issues is greater among users than nonusers, and in particular, the burden of mental illness is much greater on users compared to nonusers. However, greater usage does not translate into higher perceived usefulness: participants considering SCs useful were more commonly the healthier users and especially less often users reporting to suffer from mental health problems or undergo psychotherapy. The high burden of mental health issues among users highlights the importance of studying the effects of mental health on usability, perceived usefulness, and risks of SC usage.

##### Affinity for Technology Interaction and Self-Efficacy

An affinity for technology interaction increased the likelihood of knowing about SCs and finding them useful, but it had no effect on the intention to use SCs. Lastly, higher self-efficacy was associated with a greater likelihood of finding SCs useful but not with knowledge about them or the intention to use them. The visual inspection of the data leads us to hypothesize a nonlinear relationship between an affinity for technology interaction, self-efficacy, and considering SCs useful: Individuals below a certain threshold of self-efficacy (which is situated below the population’s average score) might likely not report finding such tools useful. In contrast, individuals above a certain threshold of affinity for technology (which is situated far above the population’s average score) are highly likely to consider SCs useful.

### Limitations

We used a sample stratified by gender, state of residence, net household income, and age. However, since we used an online questionnaire, there was a risk of selection bias—choosing people who had a higher affinity for technology than the general population. Franke et al [46] found differences in the affinity for technology between samples recruited online and offline in a validation study. A university and social media sample had a mean affinity for technology of 4.14, while a random sample in German cities (using pen and paper) had a mean affinity for technology of 3.58. Our study sample’s average ATI score was 3.74 and thus slightly higher than expected for a random sample among both “offliners” and “onliners.” As most of our participants (>91.9%) indicated using the internet multiple times a day, we certainly missed the population subgroup with low technological affinity.

Although the sample size was suitable for the aim of this work, the subsets of those aware of and those using SCs were rather small. Thus, we might have missed important associations of a smaller effect size and overestimated the degree of other associations by chance. Due to the nature of exploratory analyses, which investigate a multitude of associations at once, our findings are subject to the multiple testing problem. However, we conducted robustness checks correcting *P* values. Although we replicated findings from previous studies (eg, users being more often female, younger, and well educated), other results, such as men finding SCs more useful than women, need to be replicated in future studies. Especially, the hypotheses we derived from the presented findings must be replicated in further studies—for example, that a lack of awareness is the driver behind people’s intention to use SCs, rather than sociodemographic differences.

Our data stem from a cross-sectional online survey of the German population. Thus, all the data are self-reported data. Especially concerning mental health, we might have missed participants with a mental illness who are not open to reporting this in a survey.

As a final point, we evaluated only the subjective usefulness of SCs, not their objective usefulness (eg, facilitating safer, more informed decision-making and guiding users toward appropriate health care facilities). SCs were, for example, perceived as more useful by men, but whether they led to better decisions or other favorable outcomes remains unproven.

### Conclusion

Our findings hint at a misalignment between factors associated with using and benefiting from SCs. That is, some groups could potentially benefit from these tools but are uninclined to use them or are unaware of them, while other groups could be inclined to use SCs but seem to not benefit from them. Based on these observed misalignments, our data suggest that SCs currently fail to alleviate inequalities in access to health care despite their high availability and convenient service: Users with higher educational levels, income, and self-efficacy are more likely to report benefiting from SCs, while users with lower self-reported health and mental health issues are less likely to do so. Simply expanding the awareness of SCs among the general population to reduce unequal awareness and unequal usage might leave this unequal distribution of benefits intact.

Ultimately, how the outlined misalignments between using and benefiting from SCs will evolve when a greater part of the general population becomes familiar with SCs and decides to use them remains an open question.

Our findings provide some indication that SCs’ full potential—in terms of users considering them useful—might be tapped by the majority and late adopters (ie, those adopting an innovation after the commonly younger and more educated [68,69] innovators and early adopters). However, health variables unrelated to the inclination to adopt technology early—such as the specific medical concern, past medical history, and the context an SC is approached with—are associated with perceived usefulness and might be a bigger factor influencing whether an SC offers its user a valuable service. To get consumers and patients who are more likely to benefit from SCs to use them, they must have a low-barrier point of contact in the patient journey. Thus, integrating them into the standard health care system might prove a more fruitful path forward than simply promoting (or discouraging) the stand-alone use of such apps.

## Appendix

Multimedia Appendix 1 Full tables and additional analyses.

## Abbreviations

ATI: Affinity for Technology Interaction
OR: odds ratio
SC: symptom checker
