# Supplementary material for: Characteristics of Users and Nonusers of Symptom Checkers in Germany: Cross-Sectional Survey Study
Source: J Med Internet Res. 2023 Jun 20;25:e46231. doi: 10.2196/46231 (PMC10337374; doi:10.2196/46231)

Table S1. All Characteristics and Inter-individual Differences of Respondents Knowing Symptom Checkers and Those Not Knowing.

| Variable | | | Aware of Symptom Checkers | Not aware of Symptom Checkers | Inferential Statistics |
| --- | --- | --- | --- | --- | --- |
| N (%)  [95% Confidence Interval %] | | | 177 (16.3%)  [14.2 – 18.7%] | 907 (83.7%)  [81.3 – 85.8%] | / |
| Age, M (SD) | | | 38.8 (14.6) | 48.3 (15.7) | t(265.27) = 7.93,  P < .001*,  d = 0.63 |
| Gender, n (%)  [95% Confidence Interval %] | | |  |  | P = .015*,  V = 0.08 |
|  | Male | | 69 (39.0%)  [32.2 – 46.8%] | 452 (49.8%)  [46.5 - 53.3%] |  |
|  | Female | | 107 (60.5%)  [53.7 – 68.3%] | 453 (49.9%)  [46.6 - 53.4%] |  |
|  | Diverse | | 1 (0.6%)  [0.0 - 8.4%] | 2 (0.2%)  [0.0 - 3.7%] |  |
| Education, n (%)  [95% Confidence Interval %] | | |  |  | P <.001*,  V = 0.150 |
|  | No school diploma | | 0 (0.0%)  [0.0 - 7.8%] | 5 (0.6%)  [0 – 4.0%] |  |
|  | Primary school / lower secondary school | | 7 (4.0%)  [0.0 - 11.8%] | 54 (6.0%)  [2.5 - 9.4%] |  |
|  | Secondary school leaving certificate | | 27 (15.3%)  [7.9 - 23.1%] | 198 (21.8%)  [18.4 - 25.3%] |  |
|  | A levels / high school diploma | | 35 (19.8%)  [12.4 - 27.6%] | 131 (14.4%)  [11.0 - 17.9%] |  |
|  | Completed vocational training | | 36 (20.3%)  [13.0 - 28.2%] | 281 (31.0%)  [27.6 - 34.4%] |  |
|  | University or college degree | | 72 (40.7%)  [33.3 - 48.5%] | 238 (26.2%)  [22.8 - 29.7%] |  |
| Monthly Net Household Income in €, M (SD) | | | 2174 (993) | 1814 (865) | t(224.1) = 4.40,  P < .001*,  d = 0.387 |
| Municipality size, n of residents (%) [95% Confidence Interval %] | | |  |  | χ2 (12) = 11.48,  P = .489 |
|  | Less than 5.000 | | 21 (11.9%)  [5.1 - 18.8%] | 157 (17.3%)  [14.2 - 20.4%] |  |
|  | 5.000 – 10.000 | | 25 (14.1%)  [7.3 - 21.0%] | 104 (11.5%)  [8.4 - 14.6%] |  |
|  | 10.000 – 20.000 | | 22 (12.4%)  [5.6 - 19.3%] | 124 (13.7%)  [10.6 - 16.8%] |  |
|  | 20.000 – 50.000 | | 31 (17.5%)  [10.7 - 24.4%] | 137 (15.1%)  [12.0 - 18.2%] |  |
|  | 50.000 – 100.000 | | 18 (10.2%)  [3.4 - 17.1%] | 83 (9.2%)  [6.1 - 12.2%] |  |
|  | 100.000 – 500.000 | | 29 (16.4%)  [9.6 - 23.3%] | 167 (18.4%)  [15.3 - 21.5%] |  |
|  | More than 500.000 | | 31 (17.5%)  [10.7 - 24.4%] | 135 (14.9%)  [11.8 - 18.0%] |  |
| Migration background, n (%)  [95% Confidence Interval %] | | | 23 (13.1%)  [8.9 - 18.8%] | 100 (11.0%)  [9.1 - 13.2%] | χ2 (1) = 0.437,  P = .509 |
| Native German speaker, n (%)  [95% Confidence Interval %] | | | 171 (96.6%)  [92.8 - 98.4%] | 873 (96.3%)  [94.8 - 97.3%] | χ2 (1) < 0.001,  P > .99 |
| Self-efficacy, M (SD)^1^ | | | 4.03 (0.73) | 3.95 (0.71) | t(247.38) = 1.22,  P = .225 |
| General health, n (%)  [95% Confidence Interval %] | | |  |  | U (n_1_ = 177, n_2_ = 907) = 69868,  P = .003*,  rg = 0.13 |
|  | | Very bad | 2 (1.1%)  [0.0 - 8.9%] | 13 (1.4%)  [0.0 - 4.9%] |  |
|  | | Bad | 10 (5.6%)  [0.0 – 13.4%] | 87 (9.6%)  [6.3 – 13.1%] |  |
|  | | Fair | 43 (24.3%)  [16.9 - 32.1%] | 258 (28.4%)  [25.1 - 31.9%] |  |
|  | | Good | 89 (50.3%)  [42.9 – 58.1%] | 451 (49.7%)  [46.4 – 53.2%] |  |
|  | | Very good | 33 (18.6%)  [11.3 – 26.4%] | 98 (10.8%)  [7.5 - 14.3%] |  |
| Restrictions for health reasons, n (%)  [95% Confidence Interval %] | | |  |  | U (n_1_ = 177, n_2_ = 907) = 77176,  P = .375 |
|  | | Not limited at all | 70 (39.5%)  [32.2 –47.8%] | 389 (42.9%)  [39.5 – 46.5%] |  |
|  | | Limited but not severely | 80 (45.2%)  [37.9 – 53.4%] | 395 (43.6%)  [40.1 – 47.1%] |  |
|  | | Severely limited | 27 (15.3%)  [7.9 – 23.5 %] | 123 (13.6%)  [10.1 – 17.1%] |  |
| Chronic disease, n (%)  [95% Confidence Interval %] | | | 83 (46.9%)  [39.7 - 54.2%] | 461 (50.8%)  [47.6 - 54.1%] | χ2 (1) = 0.701,  P = .402 |
| Depression, n (%)  [95% Confidence Interval %] | | | 31 (17.5%)  [12.6 - 23.8%] | 135 (14.9%)  [12.7 - 17.3%] | χ2 (1) = 0.678,  P = .410 |
| Panic or anxiety disorder, n (%)  [95% Confidence Interval %] | | | 27 (15.3%)  [10.7 - 21.3%] | 83 (9.2%)  [7.4 - 11.2%] | χ2 (1) = 5.40,  P = .020*,  φ = 0.07 |
| Chronic pain, n (%)  [95% Confidence Interval %] | | | 24 (13.6%)  [9.3 - 19.4%] | 117 (12.9%)  [10.9 - 15.2%] | χ2 (1) = 0.004,  P = .948 |
| Type of health insurance, n(%)  [95% Confidence Interval %] | | |  |  | P = .075 |
|  | Without health insurance | | 0 (0.0%)  [0.0 - 5.4%] | 4 (0.4%)  [0.0 - 2.4%] |  |
|  | Statutory health insurance | | 147 (83.1%)  [78.0 - 88.5%] | 811 (89.5%)  [87.5 - 91.4%] |  |
|  | Private health insurance | | 29 (16.4%)  [11.3 - 21.8%] | 85 (9.4%)  [7.5 - 11.3%] |  |
|  | Other | | 1 (0.5%)  [0.0 - 6.0%] | 6 (0.7%)  [0 – 2.6%] |  |
| Permanent general practitioner, n(%) [95% Confidence Interval %] | | | 153 (86.4%)  [80.6 - 90.7%] | 831 (91.6%)  [89.6 - 93.3%] | χ2 (1) = 4.15,  P = .042,  φ = 0.07 |
| Number of physician visits in the last year, M (SD) | | | 3.65 (3.82) | 3.91 (6.51) | t(416.53) = 0.70,  P = .485 |
| In psychotherapy, n (%)  [95% Confidence Interval %] | | | 25 (14.2%)  [9.8 - 20.1%] | 68 (7.5%)  [6.0 - 9.5%] | χ2 (1) = 7.39,  P = .007*,  φ = 0.09 |
| At least one inpatient hospital stay in the last year, n (%) [95% Confidence Interval %] | | | 36 (20.3%)  [15.1 – 26.9%] | 135 (14.9%)  [12.7 – 17.3%] | χ2 (1) = 3.086,  P = .079 |
| Frequency of internet use, n(%)  [95% Confidence Interval %] | | |  |  | P = .049,  V = 0.089 |
|  | Multiple times a day | | 170 (96.0%)  [93.8 - 98.7%] | 826 (91.1%)  [89.4 - 92.9%] |  |
|  | Once a day | | 6 (3.4%)  [1.1 - 6.1%] | 59 (6.5%)  [4.9 - 8.3%] |  |
|  | Multiple times a week | | 0 (0%)  [0.0 - 2.7%] | 19 (2.1%)  [0.4 - 3.9%] |  |
|  | Multiple times a month | | 1 (0.6%)  [0 – 3.2%] | 1 (0.1%)  [0 – 1.9%] |  |
|  | Less than once a month | | 0 (0%)  [0.0 - 2.7%] | 2 (0.2%)  [0 – 2.0%] |  |
| Affinity for technology, M (SD)^2^ | | | 4.17 (0.93) | 3.66 (0.99) | t(264.12) = 6.51,  P < .001*,  d = 0.53 |
| General health app usage, n (%) [95% Confidence Interval %] | | | 128 (74.0%)  [67.0 – 80.0%] | 389 (43.5%)  [40.3 - 46.8%] | χ2 (2) = 54.03,  P < .001*,  φ = 0.22 |

Note: Some p-values are reported without a test statistic as we used a Fisher’s exact test. For factors with multiple factor levels (eg, gender and education) we report summary test statistics from a Chi-Square test / Fisher’s exact test, that is they pertain to the differences of the distribution of factor levels between the two groups and not to group differences regarding single factor levels (eg, “Male”, “No school diploma”).

^1^ On a scale from 1 to 5

^2^ On a scale from 1 to 6

* Result remains statistically significant when correcting for multiple testing using the Benjamini-Hochberg procedure

Table S2. All Characteristics and Inter-individual Differences of Symptom Checker Users and Non-Users.

| Variable | | | Users | Non-Users | Inferential Statistics |
| --- | --- | --- | --- | --- | --- |
| N (%),  [95% Confidence Interval %] | | | 71 (6.5%)  [5.2 – 8.2%] | 1013 (93.5%)  [91.8 – 94.9%] | / |
| Age, M (SD) | | | 37.6 (14.3) | 47.3 (15.8) | t(82.56) = 5.52,  P < .001*,  d = 0.65 |
| Gender^1^, n (%)  [95% Confidence Interval %] | | |  |  | P = .030,  V = 0.08 |
|  | Male | | 26 (36.62%)  [26.8 - 49.0%] | 495 (48.86%)  [45.7 - 52.1%] |  |
|  | Female | | 44 (61.97%)  [52.1 - 74.4%] | 516 (50.94%)  [47.8 - 54.2%] |  |
|  | Diverse | | 1 (1.41%)  [0.0 - 13.8%] | 2 (0.20%)  [0.0 - 3.5%] |  |
| Education, n (%)  [95% Confidence Interval %] | | |  |  | P = .035,  V = 0.104 |
|  | No school diploma | | 0 (0%)  [0.0 - 12.2%] | 5 (0.49%)  [0.0 - 3.8%] |  |
|  | Primary school / lower secondary school | | 4 (5.63%)  [0.0 - 17.8%] | 57 (5.63%)  [2.5 - 9.0%] |  |
|  | Secondary school leaving certificate | | 7 (9.86%)  [0.0 - 22.0%] | 218 (21.52%)  [18.4 - 24.9%] |  |
|  | (Fach-)Abitur (A levels / high school diploma) | | 14 (19.72%)  [8.5 - 31.9%] | 152 (15.00%)  [11.8 - 18.3%] |  |
|  | Completed vocational training | | 16 (22.54%)  [11.3 - 34.7%] | 301 (29.71%)  [26.6 - 33.1%] |  |
|  | University or college degree | | 30 (42.25%)  [31.0 - 54.4%] | 280 (27.64%)  [24.5 - 31.0%] |  |
| Monthly Net Household Income in €, M (SD) | | | 2248 (1053) | 1841 (876) | t(76.132) = 3.16,  P = .002*,  d = 0.42 |
| Municipality size, n of residents (%)  [95% Confidence Interval %] | | |  |  | χ2 (6) = 7.54,  P = .274 |
|  | Less than 5.000 | | 7 (9.86%)  [0.0 - 21.8%] | 171 (16.88%)  [14.0 - 19.9%] |  |
|  | 5.000 – 10.000 | | 10 (14.08%)  [4.2 - 26.0%] | 119 (11.75%)  [8.9 - 14.7%] |  |
|  | 10.000 – 20.000 | | 7 (9.86%)  [0.0 - 21.8%] | 139 (13.72%)  [10.9 - 16.7%] |  |
|  | 20.000 – 50.000 | | 8 (11.27%)  [1.4 - 23.2%] | 160 (15.79%)  [12.9 - 18.8%] |  |
|  | 50.000 – 100.000 | | 7 (9.86%)  [0.0 - 21.8%] | 94 (9.28%)  [6.4 - 12.3%] |  |
|  | 100.000 – 500.000 | | 19 (26.76%)  [16.9 - 38.7%] | 177 (17.47%)  [14.6 - 20.5%] |  |
|  | More than 500.000 | | 13 (18.31%)  [8.5 - 30.2%] | 153 (15.10%)  [12.2 - 18.1%] |  |
| Migration background, n (%)  [95% Confidence Interval %] | | | 8 (11.27%)  [5.8 - 20.7%] | 115 (11.35%)  [9.5 - 13.5%] | χ2 (1) < 0.001,  P > .99 |
| Native German speaker, n (%)  [95% Confidence Interval %] | | | 69 (97.18%)  [90.3 - 99.2%] | 975 (96.25%)  [9.5 - 13.5%] | χ2 (1) = 0.006,  P = .938 |
| Self-efficacy, M (SD)^1^ | | | 4.01 (0.68) | 3.96 (0.72) | t(81.33) = 0.66,  P = .511 |
| General health, n (%)  [95% Confidence Interval %] | | | 3.61 (0.82) | 3.62 (0.86) | U (n_1_ = 71, n_2_ = 1013) = 36626,  P = .778 |
|  | | Very bad | 1 (1.4%)  [0.0 - 13.5%] | 14 (1.4%)  [0.0 - 4.7%] |  |
|  | | Bad | 5 (7.0%)  [0.0 – 19.2%] | 92 (9.1%)  [5.9 – 12.4%] |  |
|  | | Fair | 22 (31.0%)  [19.7 - 43.1%] | 279 (27.5%)  [24.4 - 30.8%] |  |
|  | | Good | 36 (50.7%)  [39.4 – 62.8%] | 504 (49.8%)  [46.6 – 53.0%] |  |
|  | | Very good | 7 (9.9%)  [0.0 – 22.0%] | 124 (12.2%)  [9.1 - 15.5%] |  |
| Restrictions for health reasons, n (%)  [95% Confidence Interval %] | | |  |  | U (n_1_ = 71, n_2_ = 1013) = 28556,  P = .002*,  rg = .20 |
|  | | Not limited at all | 17 (23.9%)  [12.7 –35.7%] | 442 (43.6%)  [40.4 – 47.0%] |  |
|  | | Limited but not severely | 40 (56.3%)  [45.1 – 68.1%] | 435 (42.9%)  [39.7 – 46.3%] |  |
|  | | Severely limited | 14 (19.7%)  [8.5 – 31.5 %] | 136 (13.4%)  [10.2 – 16.8%] |  |
| Chronic disease, n (%)  [95% Confidence Interval %] | | | 41 (57.75%)  [46.2 - 68.5%] | 503 (49.65%)  [46.6 - 52.7%] | χ2 (1) = 1.429,  P = 0.232 |
| Depression, n (%)  [95% Confidence Interval %] | | | 22 (30.99%)  [21.4 - 42.5%] | 144 (14.22%)  [12.2 - 16.5%] | χ2 (1) = 13.13,  P < .001*,  φ = 0.12 |
| Panic or anxiety disorder, n (%)  [95% Confidence Interval %] | | | 16 (22.54%)  [14.4 - 33.5%] | 94 (9.28%)  [7.6 - 11.2%] | χ2 (1) = 11.37,  P < .001*,  φ = 0.11 |
| Chronic pain, n (%)  [95% Confidence Interval %] | | | 14 (19.72%)  [12.1 - 30.4%] | 127 (12.54%)  [10.6 - 14.7%] | χ2 (1) = 2.423,  P = .120 |
| Type of health insurance, n (%)  [95% Confidence Interval %] | | |  |  | P = .248 |
|  | Without health insurance | | 0 (0%)  [0.0 - 9.6%] | 4 (0.40%)  [0.0 - 2.3%] |  |
|  | Statutory health insurance | | 58 (81.69%)  [74.6 - 91.3%] | 900 (88.93%)  [87.2 - 90.9%] |  |
|  | Private health insurance | | 13 (18.31%)  [11.3 - 27.9%] | 101 (9.98%)  [8.2 - 11.9%] |  |
|  | Other | | 0 (0%)  [0.0 - 9.6%] | 7 (0.69%)  [0.0 - 2.6%] |  |
| Permanent general practitioner, n (%)  [95% Confidence Interval %] | | | 67 (94.4%) [86.4 - 97.8%] | 917 (90.5%) [88.6 - 92.2%] | χ2 (1) = 0.756,  P = .385 |
| Number of physician visits in the last year, M (SD) | | | 4.51 (3.69) | 3.82 (6.30) | t(101.08) = 1.43,  P = .157 |
| Currently in psychotherapy, n (%)  [95% Confidence Interval %] | | | 18 (25.4%)  [16.7 - 36.6%] | 75 (7.5%)  [5.9 - 9.2%] | χ2 (1) = 24.62,  P < .001*,  φ = 0.16 |
| At least one inpatient hospital stay in the last year, n (%)  [95% Confidence Interval %] | | | 21 (29.6%)  [20.2 - 41.0%] | 150 (14.8%)  [12.8 - 17.1%] | χ2 (1) = 10.12,  P = .002*,  φ = 0.10 |
| Frequency of internet use, n (%)  [95% Confidence Interval %] | | |  |  | P = .527 |
|  | Multiple times a day | | 69 (97.2%)  [94.4 - 100.0%] | 927 (91.51%) [89.9 - 93.1%] |  |
|  | Once a day | | 2 (2.82%)  [0.0 - 5.7%] | 63 (6.22%)  [4.6 - 7.8%] |  |
|  | Multiple times a week | | 0 (0%)  [0.0 - 2.9%] | 19 (1.88%)  [0.3 - 3.5%] |  |
|  | Multiple times a month | | 0 (0%)  [0.0 - 2.9%] | 2 (0.20%)  [0.0 - 1.8%] |  |
|  | Less than once a month | | 0 (0%)  [0.0 - 2.9%] | 2 (0.20%)  [0.0 - 1.8%] |  |
| Affinity for technology, M (SD)^2^ | | | 4.13 (0.95) | 3.72 (1.00) | t(81.16) = 3.49,  P < .001*,  d = 0.42 |
| General health app usage, n (%)  [95% Confidence Interval %] | | | 61 (85.92%)  [76.0 - 92.2%] | 456 (45.74%)  [42.0 - 48.1%] | χ2 (1) = 43.26,  P < .001*,  φ = 0.21 |

Note: Some p-values are reported without a test statistic because of the Fisher’s exact test. Factors with multiple factor levels were tested combined using a Chi-Square test / Fisher’s exact test.

^1^ On a scale from 1 to 5

^2^ On a scale from 1 to 6

* Result remains statistically significant when correcting for multiple testing using the Benjamini-Hochberg procedure

Table S3. All Characteristics and Interindividual Differences of Symptom Checker Users and Non-Users Among Those Knowing Symptom Checkers.

| Variable | | | Uses Symptom Checker | Aware of but not using symptom checker |  |
| --- | --- | --- | --- | --- | --- |
| N (%)  [95% Confidence Interval %] | | | 71 (40.1%)  [32.8 – 47.7%] | 106 (59.9%)  [52.3 – 67.2%] | / |
| Age, M (SD) | | | 37.6 (14.3) | 39.6 (14.8) | t(153.74) = 0.88,  P = .380 |
| Gender, n (%)  [95% Confidence Interval %] | | |  |  | P = .464 |
|  | Male | | 26 (36.6%)  [26.8 - 49.0%] | 43 (40.6%)  [32.1 - 50.8%] |  |
|  | Female | | 44 (62.0%)  [52.1 – 74.4%] | 63 (59.4%)  [50.9 - 69.7%] |  |
|  | Diverse | | 1 (1.4%)  [0.0 – 13.8%] | 0 (0.0%)  [0.0 – 0.0%] |  |
| Education, n (%)  [95% Confidence Interval %] | | |  |  | P = .478 |
|  | No school diploma | | 0 (0.0%)  [0.0 – 12.2%] | 0 (0.0%)  [0.0 – 10.0%] |  |
|  | Primary school / lower secondary school | | 4 (5.6%)  [0.0 – 17.8%] | 3 (2.8%)  [0.0 – 12.9%] |  |
|  | Secondary school leaving certificate | | 7 (9.9%)  [0.0 – 22.0%] | 20 (18.9%)  [9.4 – 28.9%] |  |
|  | A levels / high school diploma | | 14 (19.7%)  [8.5 – 31.9%] | 21 (19.8%)  [10.4 – 29.8%] |  |
|  | Completed vocational training | | 16 (22.5%)  [11.3 – 34.7%] | 20 (18.9%)  [9.4 – 28.9%] |  |
|  | University or college degree) | | 30 (42.3%)  [31.0 – 54.4%] | 42 (39.6%)  [30.2 – 49.6%] |  |
| Monthly Net Household Income in €, M (SD) | | | 2248 (1053) | 2122 (951) | t(138.77) = 0.80,  P = .425 |
| Municipality size, n of residents (%)  [95% Confidence Interval %] | | |  |  | χ2 (6) = 11.52,  P = .074 |
|  | Less than 5.000 | | 7 (9.9%)  [0.0 – 21.8%] | 14 (13.2%)  [4.7 – 22.4%] |  |
|  | 5.000 – 10.000 | | 10 (14.1%)  [4.2 – 26.0%] | 15 (14.2%)  [5.7 – 23.4%] |  |
|  | 10.000 – 20.000 | | 7 (9.9%)  [0.0 – 21.8%] | 15 (14.2%)  [5.7 – 23.4%] |  |
|  | 20.000 – 50.000 | | 8 (11.3%)  [1.4 – 23.2] | 23 (21.7%)  [13.2 – 30.9%] |  |
|  | 50.000 – 100.000 | | 7 (9.9%)  [0.0 – 21.8%] | 11 (10.4%)  [1.9 – 19.6%] |  |
|  | 100.000 – 500.000 | | 19 (26.8%)  [16.9 – 38.7%] | 10 (9.4%)  [0.9 – 18.6%] |  |
|  | More than 500.000 | | 13 (18.3%)  [8.5 – 30.2%] | 18 (17.0%)  [8.5 – 26.2%] |  |
| Migration background, n (%)  [95% Confidence Interval %] | | | 8 (11.3%)  [5.8 – 20.7%] | 15 (14.2%)  [8.8 – 22.0%] | χ2 (1) = 0.13,  P = .723 |
| Native German speaker, n (%)  [95% Confidence Interval %] | | | 69 (97.2%)  [90.3 – 99.2%] | 102 (96.2%)  [90.7 – 98.5%] | χ2 (1) < 0.001,  P > .999 |
| Self-efficacy, M (SD)^1^ | | | 4.01 (0.68) | 4.04 (0.76) | t(160.87) = 0.25,  P = .806 |
| General health, n (%)  [95% Confidence Interval %] | | |  |  |  |
|  | | Very bad | 1 (1.4%)  [0.0 - 13.5%] | 1 (0.9%)  [0.0 - 11.0%] | U (n_1_ = 71, n_2_ = 106) = 4568,  P = .009*,  rg = .21 |
|  | | Bad | 5 (7.0%)  [0.0 – 19.2%] | 5 (4.7%)  [0.0 – 14.7%] |  |
|  | | Fair | 22 (31.0%)  [19.7 - 43.1%] | 21 (19.8%)  [10.4 - 29.8%] |  |
|  | | Good | 36 (50.7%)  [39.4 – 62.8%] | 53 (50.0%)  [40.6 – 60.0%] |  |
|  | | Very good | 7 (9.9%)  [0.0 – 22.0%] | 26 (24.5%)  [15.1 - 34.5%] |  |
| Restrictions for health reasons, n (%)  [95% Confidence Interval %] | | |  |  |  |
|  | | Not limited at all | 17 (23.9%)  [12.7 –35.7%] | 53 (50.0%)  [40.6 – 60.2%] | U (n_1_ = 71, n_2_ = 106) = 2762,  P = .001*,  rg = .27 |
|  | | Limited but not severely | 40 (56.3%)  [45.1 – 68.1%] | 40 (39.6%)  [28.3 – 47.9%] |  |
|  | | Severely limited | 14 (19.7%)  [8.5 – 31.5 %] | 13 (12.3%)  [2.8 – 22.5%] |  |
| Chronic disease, n (%)  [95% Confidence Interval %] | | | 41 (57.7%)  [46.2 – 68.5%] | 42 (39.6%)  [30.8 – 49.1%] | χ2 (1) = 4.90,  P = .027,  φ = 0.18 |
| Depression, n (%)  [95% Confidence Interval %] | | | 22 (31.0%)  [21.4 – 42.5%] | 9 (8.5%)  [4.5 – 15.4] | χ2 (1) = 13.38,  P < .001*,  φ = 0.29 |
| Panic or anxiety disorder, n (%)  [95% Confidence Interval %] | | | 16 (22.5%)  [14.4 – 33.5%] | 11 (10.4%)  [5.9 – 17.6%] | χ2 (1) = 3.97,  P = .046,  φ = 0.17 |
| Chronic pain, n (%)  [95% Confidence Interval %] | | | 14 (19.7%)  [12.1 – 30.4%] | 10 (9.4%)  [5.2 – 16.5%] | χ2 (1) = 3.0,  P = .083 |
| Type of health insurance, n(%)  [95% Confidence Interval %] | | |  |  | P = .808 |
|  | Without health insurance | | 0 (0.0%)  [0.0 – 9.6%] | 0 (0.0%)  [0.0 – 7.3%] |  |
|  | Statutory health insurance | | 58 (81.7%)  [74.6 – 91.3%] | 89 (84.0%)  [78.3 – 91.3%] |  |
|  | Private health insurance | | 13 (18.3%)  [11.3 – 27.9%] | 16 (15.1%)  [9.4 – 22.4%] |  |
|  | Other | | 0 (0.0%)  [0.0 – 9.6%] | 1 (0.9%)  [0.0 – 8.3%] |  |
| Permanent general practitioner, n(%) [95% Confidence Interval %] | | | 67 (94.4%)  [86.4 – 97.8%] | 86 (81.1%)  [72.6 – 87.4%] | χ2 (1) = 5.27,  P = .022,  φ = 0.19 |
| Number of physician visits in the last year, M (SD) | | | 4.51 (3.69) | 3.08 (3.81) | t(153.43) = 2.50,  P = .014,  d = 0.382 |
| Currently in psychotherapy, n (%)  [95% Confidence Interval %] | | | 18 (25.4%)  [16.7 – 36.6%] | 7 (6.6%)  [3.2 – 13.0%] | χ2 (1) = 10.65,  P = .001*,  φ = 0.26 |
| At least one inpatient hospital stays in the last year, n (%) [95% Confidence Interval %] | | | 21 (29.6%)  [20.2 – 41.0%] | 15 (14.2%)  [8.8 – 22.0%] | χ2 (1) = 5.33,  P = .021,  φ = 0.19 |
| Frequency of internet use, n (%)  [95% Confidence Interval %] | | |  |  | P > .999 |
|  | Multiple times a day | | 69 (97.2%)  [94.4 - 1.0%] | 101 (95.3%)  [92.5 – 99.2%] |  |
|  | Once a day | | 2 (2.8%)  [0.0 – 5.7%] | 4 (3.8%)  [0.9 – 7.7%] |  |
|  | Multiple times a week | | 0 (0.0%)  [0.0 – 2.9%] | 0 (0.0%)  [0.0 – 4.0%] |  |
|  | Multiple times a month | | 0 (0.0%)  [0.0 – 2.9%] | 1 (0.9%)  [0.0 – 4.9%] |  |
|  | Less than once a month | | 0 (0.0%)  [0.0 – 2.9%] | 0 (0.0%)  [0.0 – 4.0%] |  |
| Affinity for technology, M (SD)^2^ | | | 4.13 (0.95) | 4.19 (0.91) | t(145.94) = 0.49,  P = .627 |
| General health app usage, n (%)  [95% Confidence Interval %] | | | 61 (85.9%)  [76.0 – 92.2%] | 67 (63.2%)  [53.7 – 71.8%] | χ2 (1) = 9.45,  P = .002*,  φ = 0.25 |

Note: Some p-values are reported without a test statistic because of the Fisher’s exact test. Factors with multiple factor levels were tested combined using a Chi-Square test / Fisher’s exact test.

^1^ On a scale from 1 to 5

^2^ On a scale from 1 to 6

* Result remains statistically significant when correcting for multiple testing using the Benjamini-Hochberg procedure

Table S4. All Characteristics and Interindividual Differences of Users Considering Symptom Checkers Useful and Those Who Do Not

| Variable | | | Considered Symptom Checker Useful | Considered Symptom Checker Sometimes Useful, Sometimes Not | Did Not Consider Symptom Checker Useful | Inferential statistics Comparing “Considered Useful” and “Did Not Consider Useful” |
| --- | --- | --- | --- | --- | --- | --- |
| N | | | 29 (40.8%)  [29.3 – 53.2%] | 24 (33.8%)  [23.0 – 46.0%] | 18 (25.4%)  [15.8 – 37.1%] | / |
| Age, M (SD) | | | 38.6 (13.4) | 36.2 (15.3) | 37.9 (14.9) | t(33.40) = 0.15,  P = .882 |
| Gender, n (%)  [95% Confidence Interval %] | | |  |  |  | P = .044,  V = 0.356 |
|  | Male | | 16 (55.2%)  [41.4 – 75.8%] | 6 (25.0%)  [12.5 – 43.9%] | 4 (22.2%)  [5.6 – 42.4%] |  |
|  | Female | | 13 (44.8%)  [31.0 – 65.5%] | 18 (75.0%)  [62.5 – 93.9%] | 13 (72.2%)  [55.6 – 92.4%] |  |
|  | Diverse | | 0 (0.0%)  [0.0 – 20.6%] | 0 (0.0%)  [0.0 – 18.9%] | 1 (5.6%)  [0.0 – 25.7%] |  |
| Education, n (%)  [95% Confidence Interval %] | | |  |  |  | P = .016,  V = 0.493 |
|  | No school diploma | | 0 (0.0%)  [0.0 – 19.7%] | 0 (0.0%)  [0.0 – 22.3%] | 0 (0.0%)  [0.0 – 27.7%] |  |
|  | Primary school / lower secondary school | | 2 (6.9%)  [0.0 – 26.6%] | 1 (4.2%)  [0.0 – 26.45%] | 1 (5.6%)  [0.0 – 33.2%] |  |
|  | Secondary school leaving certificate | | 4 (13.8%)  [0.0 – 33.5%] | 3 (12.5%)  [0.0 – 34.8%] | 0 (0.0%)  [0.0 – 27.7%] |  |
|  | A levels / high school diploma | | 3 (10.3%)  [0.0 – 30.1%] | 5 (20.8%)  [4.2 – 43.1%] | 6 (33.3%)  [16.7 – 61.0%] |  |
|  | Completed vocational training | | 4 (13.8%)  [0.0 – 33.5%] | 5 (20.8%)  [4.2 – 43.1%] | 7 (38.9%)  [22.2 – 66.5%] |  |
|  | University or college degree | | 16 (55.2%)  [41.4 – 74.9%] | 10 (41.7%)  [25.0 – 64.0%] | 4 (22.2%)  [5.6 – 49.9%] |  |
| Monthly Net Household Income in €, M (SD) | | | 2591 (1104) | 2273 (1055) | 1627 (649) | t(43.99) = 3.74,  P < .001*,  d = 1.07 |
| Municipality size, n of residents (%)  [95% Confidence Interval %] | | |  |  |  | P = .204 |
|  | Less than 5.000 | | 1 (3.4%)  [0.0 – 23.6%] | 4 (16.7%)  [0.0 – 36.3%] | 2 (11.1%)  [0.0 – 37.3%] |  |
|  | 5.000 – 10.000 | | 5 (17.2%)  [3.4 – 37.4%] | 4 (16.7%)  [0.0 – 36.3%] | 1 (5.6)  [0.0 – 31.7%] |  |
|  | 10.000 – 20.000 | | 1 (3.4%)  [0.0 – 23.6%] | 3 (12.5%)  [0.0 – 32.1%] | 3 (16.7%)  [0.0 – 42.8%] |  |
|  | 20.000 – 50.000 | | 3 (10.3%)  [0.0 – 30.5%] | 2 (8.3%)  [0.0 – 27.9%] | 3 (16.7%)  [0.0 – 42.8%] |  |
|  | 50.000 – 100.000 | | 5 (17.2%)  [3.4 – 37.4%] | 2 (8.3%)  [0.0 – 27.9%] | 0 (0.0%)  [0.0 – 24.4%] |  |
|  | 100.000 – 500.000 | | 7 (24.1%)  [10.3 – 44.3%] | 6 (25.0%)  [8.3 – 44.6%] | 6 (33.3%)  [16.7 – 59.5%] |  |
|  | More than 500.000 | | 7 (24.1%)  [10.3 – 44.3%] | 3 (12.5%)  [0.0 – 32.1%] | 3 (16.7%)  [0.0 – 42.8%] |  |
| Migration background, n (%)  [95% Confidence Interval %] | | | 2 (6.9%)  [1.9 – 22.0%] | 4 (16.7%)  [6.7 – 35.9%] | 2 (11.1%)  [3.1 – 32.8%] | P = .631 |
| Native German speaker, n (%)  [95% Confidence Interval %] | | | 29 (100%)  [88.3 – 100.0%] | 22 (91.7%)  [74.2 – 97.7%] | 18 (100%)  [82.4 – 100.0%] | χ2 (1) < 0.001,  P > .99 |
| Self-efficacy, M (SD)^1^ | | | 4.21 (0.66) | 4.07 (0.48) | 3.63 (0.81) | t(30.70) = 2.55,  P = .016,  d = 0.78 |
| General health, n (%)  [95% Confidence Interval %] | | |  |  |  | U (n_1_ = 29, n_2_ = 18) = 185.5,  P = .071 |
|  | | Very bad | 0 (0.0%)  [0.0 - 20.3%] | 1 (4.2%)  [0.0 - 27.0%] | 0 (0.0%)  [0.0 - 27.4%] |  |
|  | | Bad | 0 (0.0%)  [0.0 - 20.3%] | 0 (4.7%)  [0.0 – 22.8%] | 5 (27.8%)  [11.1 – 55.1%] |  |
|  | | Fair | 9 (31.0%)  [17.2 - 51.4%] | 9 (37.5%)  [20.8 - 60.3%] | 4 (22.2%)  [5.6 - 50.0%] |  |
|  | | Good | 17 (58.6%)  [44.8 – 79.0%] | 11 (45.8%)  [29.2 – 68.7%] | 8 (44.4%)  [27.8 – 71.8%] |  |
|  | | Very good | 3 (10.3%)  [0.0 – 30.7%] | 3 (12.5%)  [0.0 - 35.3%] | 1 (5.6%)  [0.0 – 32.9%] |  |
| Restrictions for health reasons, n (%)  [95% Confidence Interval %] | | |  |  |  | U (n_1_ = 29, n_2_ = 18) = 304.5,  P = .295 |
|  | | Not limited at all | 8 (27.6%)  [10.3 –45.1%] | 6 (25.0%)  [8.3 – 46.1%] | 3 (16.7%)  0.0 –43.1%] |  |
|  | | Limited but not severely | 16 (55.2%)  [37.1 – 72.7%] | 14 (58.3%)  [41.7 – 79.4%] | 10 (55.6%)  [38.9 –82.0%] |  |
|  | | Severely limited | 5 (17.2%)  [0.0 – 34.7 %] | 4 (16.7%)  [0.0 – 37.7%] | 5 (27.8%)  [11.1 –54.2%] |  |
| Chronic disease, n (%)  [95% Confidence Interval %] | | | 19 (65.5%)  [47.3 – 80.1%] | 12 (50.0%)  [31.4 – 68.6%] | 10 (55.6%)  [33.7 – 75.4%] | χ2 (1) = 0.14,  P = .708 |
| Depression, n (%)  [95% Confidence Interval %] | | | 9 (31.0%)  [17.3 – 49.2] | 5 (20.8%)  [9.2 – 40.5%] | 8 (44.4%)  [24.6 – 66.3%] | χ2 (1) = 0.38,  P = .537 |
| Panic or anxiety disorder, n (%)  [95% Confidence Interval %] | | | 4 (13.8%)  [5.5 – 30.6%] | 5 (20.8%)  [9.2 – 40.5%] | 7 (38.9%)  [20.3 – 61.4%] | P = .076 |
| Chronic pain, n (%)  [95% Confidence Interval %] | | | 7 (24.1%)  [12.2 – 42.1%] | 3 (12.5%)  [4.3 – 31.0%] | 4 (22.2%)  [9.0 – 45.2%] | P > .999 |
| Type of health insurance, n(%)  [95% Confidence Interval %] | | |  |  |  | P = .034,  V = 0.330 |
|  | Without health insurance | | 0 (0.0%)  [0.0 – 14.4%] | 0 (0.0%)  [0.0 – 18.9%] | 0 (0.0%)  [0.0 – 9.0%] |  |
|  | Statutory health insurance | | 22 (75.9%)  [62.1 – 90.2%] | 18 (75.0%)  [62.5 – 93.9%] | 18 (100%)  [81.5 – 100.0%] |  |
|  | Private health insurance | | 7 (24.1%)  [10.3 – 38.5%] | 6 (25.0%)  [12.5 – 43.9%] | 0 (0.0%)  [0.0 – 9.0%] |  |
|  | Other | | 0 (0.0%)  [0.0 – 14.4%] | 0 (0.0%)  [0.0 – 18.9%] | 0 (0.0%)  [0.0 – 9.0%] |  |
| Permanent general practitioner, n(%)  [95% Confidence Interval %] | | | 29 (100%)  [88.3 – 100.0%] | 22 (91.7%)  [74.2 – 97.7%] | 16 (88.9%)  [67.2 – 96.9%] | χ2 (1) = 1.19,  P = .275 |
| Number of physician visits in the last year, M (SD) | | | 5.14 (3.36) | 3.75 (4.40) | 4.50 (3.15) | t(37.97) = 0.66,  P = .514 |
| Currently in psychotherapy, n (%)  [95% Confidence Interval %] | | | 11 (37.9%)  [22.7 – 56.0%] | 4 (16.7%)  [6.7 – 35.9%] | 3 (16.7%)  [5.8 – 39.2%] | P = .191 |
| At least one inpatient hospital stay in the last year, n(%)  [95% Confidence Interval %] | | | 12 (41.4%)  [25.5 – 59.3%] | 7 (29.2%)  [14.9 – 49.2%] | 2 (11.1%)  [3.1 – 32.8%] | P = .047  φ = 0.32 |
| Frequency of internet use, n(%)  [95% Confidence Interval %] | | |  |  |  | P > .999 |
|  | Multiple times a day | | 29 (100%)  [88.06 – 100%] | 22 (91.7%)  [87.5 – 100%] | 18 (100%)  [81.5 – 100.0%] |  |
|  | Once a day | | 0 (0.0%)  [0.0 – 5.5%] | 2 (8.3%)  [4.2 – 20.8%] | 0 (0.0%)  [0.0 – 9.0%] |  |
|  | Multiple times a week | | 0 (0.0%)  [0.0 – 5.5%] | 0 (0.0%)  [0.0 – 12.4%] | 0 (0.0%)  [0.0 – 9.0%] |  |
|  | Multiple times a month | | 0 (0.0%)  [0.0 – 5.5%] | 0 (0.0%)  [0.0 – 12.4%] | 0 (0.0%)  [0.0 – 9.0%] |  |
|  | Less than once a month | | 0 (0.0%)  [0.0 – 5.5%] | 0 (0.0%)  [0.0 – 12.4%] | 0 (0.0%)  [0.0 – 9.0%] |  |
| Affinity for technology, M (SD)^2^ | | | 4.21 (1.02) | 4.21 (0.88) | 3.86 (0.93) | t(38.79) = 1.21,  P = .233 |
| General health app usage, n (%)  [95% Confidence Interval %] | | | 25 (86.2%)  [69.4 – 94.5] | 21 (87.5%)  [69.0 – 95.7%] | 15 (83.3%)  [60.8 – 94.2] | χ2 (1) < 0.001,  P > .99 |

Note: Some p-values are reported without a test statistic because of the Fisher’s exact test. Factors with multiple factor levels were tested combined using a Chi-Square test / Fisher’s exact test.

^1^ On a scale from 1 to 5

^2^ On a scale from 1 to 6

* Result remains statistically significant when correcting for multiple testing using the Benjamini-Hochberg procedure

Figure S1. Affinity for Technology by Rating of Usefulness of Previous Symptom Checker Usage. Each dot represents a participant.


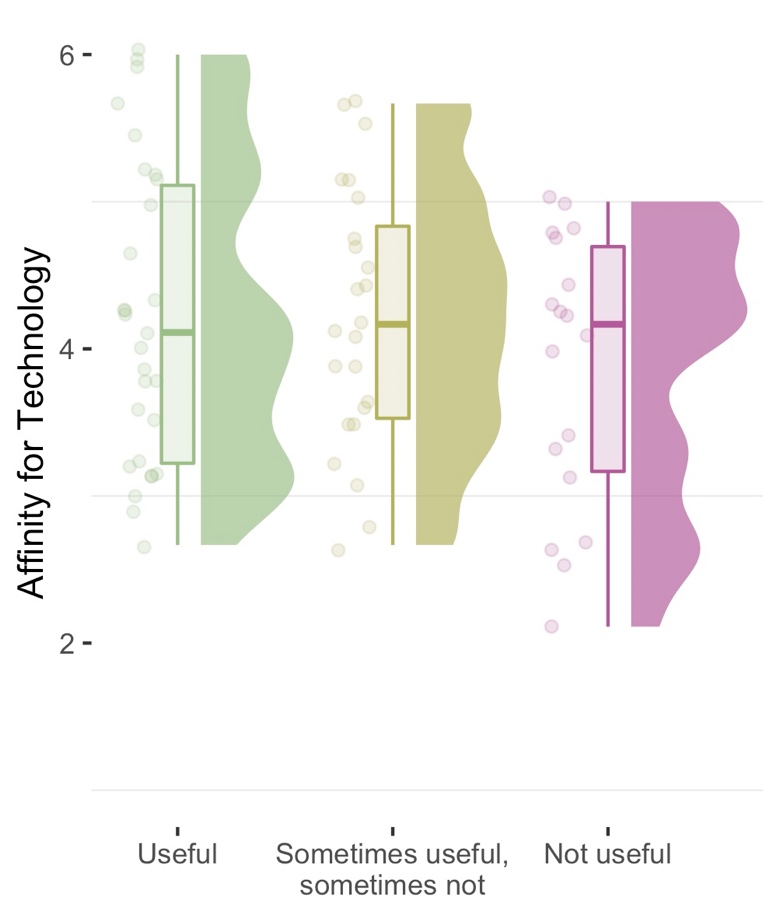


Participants who found symptom checkers useful were also more likely to have a higher income, higher self-efficacy and a better general health. The only negative associations (r  < - .10) with usefulness were panic or anxiety disorder and restrictions for health reasons.

Figure S2. Correlation Matrix of Perceived Usefulness With Other Metric and Binary Variables.


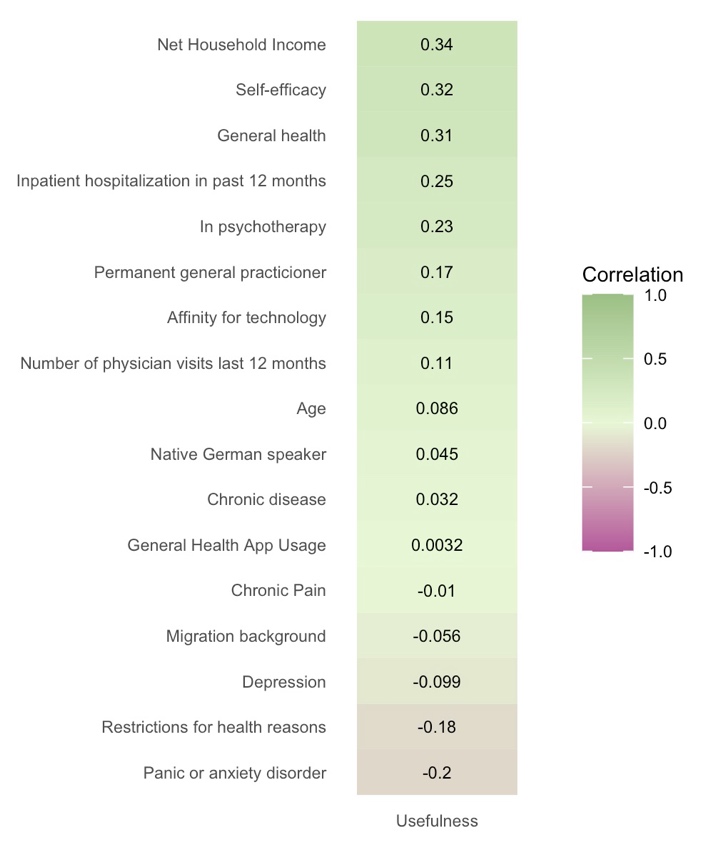

Supplement: Multimedia Appendix 1 [file jmir_v25i1e46231_app1.docx]
